# Multiscale Simulation on Product Distribution from Pyrolysis of Styrene-Butadiene Rubber

**DOI:** 10.3390/polym11121967

**Published:** 2019-11-29

**Authors:** Shengwei Deng, Han Zhuo, Yinbin Wang, Shuai Leng, Guilin Zhuang, Xing Zhong, Zhongzhe Wei, Zihao Yao, Jian-guo Wang

**Affiliations:** 1 Institute of Industrial Catalysis, College of Chemical Engineering, State Key Laboratory Breeding Base of Green-Chemical Synthesis Technology, Zhejiang University of Technology, Hangzhou 310014, China; 2 Qingdao Ecostar Intelligent Equipment Co., Ltd., Qingdao 266400, China

**Keywords:** DFT, ReaxFF MD, pyrolysis, styrene-butadiene rubber

## Abstract

Pyrolysis of styrene-butadiene rubber receives renewed attention due to its application in tackling the waste tire disposal problem while allowing energy recovery. The density functional theory calculation (DFT) and ReaxFF molecular dynamics simulation (MD) are adopted to study the pyrolysis process with the variation of temperature and pressure. The bond dissociation energies of intramonomer and intermonomer bonds in trimers with different linking methods are calculated by DFT, where the bond with low energy tends to break during the pyrolysis process. The following MD simulation shows the pyrolysis product distribution of chain segments in styrene-butadiene rubber, where bond breaking positions in MD agree well with corresponding results in DFT and experiment. The next nearest neighbor bonds (single bonds) connected with double bond or benzene usually have lower dissociation energies than other single bonds and prone to break during the pyrolysis process. And thus, the intermonomer bonds tend to break at relatively low temperatures (around 650 K in experiment) prior to intramonomer bonds, which result in the emergence of monomers. With the temperature increase, intramonomer bonds are broken and thus large fragments are further pyrolyzed into small ones (e.g., C_2_ and C). Besides, the pressure strongly influences the product distribution, where high pressures promote the occurrence of secondary reactions.

## 1. Introduction

Styrene-butadiene rubber (SBR) is one of the most versatile copolymer rubber compounds, and it is widely used in the tire production [1] where its percentage can reach up to 60%. Nowadays, the increase of waste tires causes serious environmental pollution and waste of resources [2], and the way to tackle with or recycle these waste materials become a vital issue [3]. The tire pyrolysis is considered as an efficient process for converting solid rubber waste into valuable chemicals to alleviate the environmental burdens and produces little emissions or waste during the process [4,5]. Different pyrolysis conditions lead to strong variation of product compositions [6]. Therefore, to modulate the product distribution by varying microstructures or reaction conditions is of great importance for the yield optimization of target products.

The pyrolysis process of styrene-butadiene rubber is convoluted where numerous intermediates or products are expected to form during the reaction process [7]. Based on the experimental work over the past decades, it is generally accepted that the final product distribution is the result of the coincidence of many factors such as the molecular structure [8], temperature [9], pressure [10], particle size [11], heating rate [12], carrier gas flow rate and type [13], pyrolysis time [14] and residence time of the volatiles inside the reactor [15]. The in situ experimental observations are still challenging for a deep understanding of how these variables determines the product optimization. Besides, it is difficult to perform the single factor experiment by isolating other variables. On the other hand, computer simulation develops with the rapid growth of the computer and becomes an indispensable tool for the structure and property predication of various materials [16]. Especially, computer simulation is useful in addressing the analysis of mono-factorial effects on the pyrolysis process [17], which is difficult to be achieved by experiments. 

The density functional theory (DFT) approach is extensively used to study the chemical reactions during the pyrolysis, and the calculation results are proved to be convincing by comparing to relevant experimental data [18], for example, the mechanism of phenolic pyrolysis was well explained by involving the bond dissociation energy analysis via DFT calculation [19]. For rough estimation, the self-consistent-charge DFT-tight binding (SCC-DFTB) is much less expensive than the DFT [19]. These quantum mechanics-based methods are computationally expensive that limits their applications to small systems with hundreds of atoms. For larger systems or for longer simulation time, ReaxFF molecular dynamics (MD) is developed to describe the dynamic process involving chemical reactions at the atomic level with affordable computational cost [20]. Recently, ReaxFF MD simulations are efficiently employed to investigate the reaction mechanism of pyrolysis process [19,21,22]. For example, initial reaction mechanisms of cellulose pyrolysis was revealed at different temperatures, and the simulated evolution tendencies of the major pyrolysis products agreed well with experimental observations [23]. Besides, the good agreement between the ReaxFF MD simulation results with available experimental data on the thermal decomposition of a poly(dimethylsiloxane) polymer was observed [24]. These results demonstrate that ReaxFF provides a useful computational tool for studying the chemical stability of polymers. Apparently, the combined approach of density functional theory (DFT) and molecular dynamics (MD) [25] would be effective in modeling of the decomposition processes at various reaction environments, but the effect of the rubber pyrolysis conditions on the product distribution by using this combined method only receives limited attention. 

SBR is derived from two monomers, styrene, and butadiene, which results in four kinds of repeat units in the polymer chain. The microstructures such as the compositions and linking methods are of high importance on the product distribution from pyrolysis of SBR [26]. In this work, the bond dissociation energies of all bonds in trimers are calculated by DFT with the help of Gaussian programs. To examine the difference of pyrolysis process based on repeat units, several samples are built based on four kinds of polymer chains and the ReaxFF MD simulations are performed to study the decomposition process with the temperature effects. Furthermore, the pyrolysis of styrene-butadiene rubber at difference temperatures and initial densities (pressures) are examined. The conclusion of the findings in this work is made in the final section accompanying with some prospects associated with future work.

## 2. Computational Details

### 2.1. DFT Calculations

The density functional theory calculations were carried out using the Gaussian 09 series of programs [27]. Becke 3 parameters exchange and Lee, Yang, and Parr correlation functionals [28,29] with a standard def2- TZVP basis set were used for the geometry optimizations and energy calculations, respectively.

(1) X−Y→X·+Y

(2) $$BDE=E_{X·}+E_{Y·}-E_{X-Y}$$

where the *BDE* is the bond dissociation energy of the carbon–carbon (C–C) bond in the trimers, *X* and *Y* represent atoms in both sides of the dissociated bond, *E_X·_* is the total energy of the corresponding side of dissociated bond.

**Figure 1:**
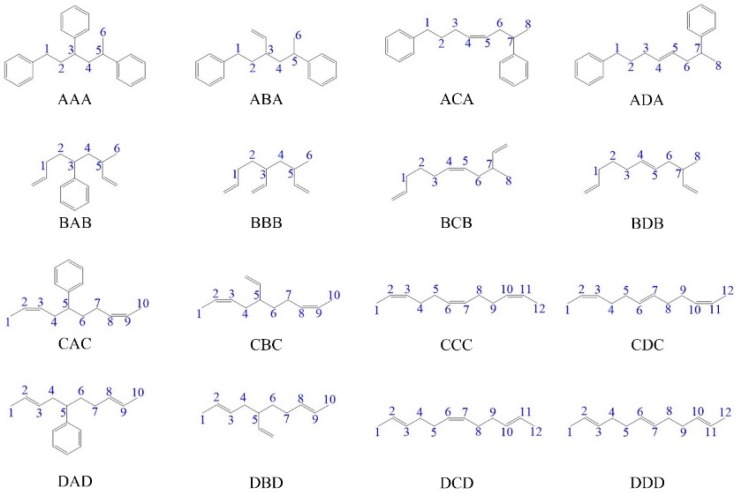
Trimers with various linking methods based on four kinds repeat units in the styrene-butadiene rubber. The C atoms in the main chain are numbered.

To represent the structure of repeat units, 16 trimers with various linking methods based on four kinds repeat units in the styrene-butadiene rubber were constructed with the help of Materials Studio Visualizer from BIOVIA. These four kinds repeat units are A: –[CH_2_–CH(C_6_H_5_)]_n_–, B: –[CH_2_–CH(CH=CH_2_)]_n_–, C (*cis*): –[CH_2_–CH=CH–CH_2_]_n_– and D (*trans*): –[CH_2_–CH=CH–CH_2_]_n_–, respectively. The number of atoms is listed in Table S1. As shown in Figure 1, all C atoms in the main chain are numbered for the distinguishment of C–C bonds.

### 2.2. ReaxFF MD Simulations

The ReaxFF can efficiently simulate the bond formation and breaking, and thus it is possible to study the chemically reactive systems [30]. This method adopts the concept of bond order to find the connectivity between atoms. The bond, angle and torsion are bond order dependent and thus these contributions are disappeared when the bond breaks. The non-bonded interactions are calculated between every pair of atoms but not limited to bonded atoms. A shielding term is included to avoid the excessive close-range nonbonded interactions. In addition, ReaxFF accounts for polarization effects via a geometry-dependent charge calculation scheme. Generally, ReaxFF uses the following equation to find the energy and then force on each atom.

(3) $$E_{system}=\;E_{bond}+E_{over}+E_{under}+E_{val}+E_{pen}+E_{tors}+E_{conj}+E_{vdWaals}+E_{Coulomb}$$

where the *E_bond_*, *E_over_*, *E_under_*, *E_val_*, *E_pen_*, *E_conj_*, *E_tors_*, *E_vdWaals_* and *E_coulomb_* represent bond energy, over-coordination energy penalty, under-coordination stability, valence angle energy, penalty energy, conjugate effect of the molecular energy, torsion angle energy, van der Waals energy and Coulomb energy, respectively. For more detail, see ref [24,31].

As a common MD force field, ReaxFF has been applied in many areas including the pyrolysis process [32]. The ReaxFF parameter set is adopted from Ref. [33], which is suitable for the study of pyrolysis process [34,35]. 14 samples are prepared based on 4 kinds of repeat units in Materials Studio as input structures, which consist of 4 homopolymer samples, 6 diblock copolymer samples and 4 SBR samples with different densities. Table S1. The number of atoms and box size for each sample are listed in Table S1. These samples were built in Materials Studio and optimized using Forcite module with the Universal Force Field (UFF) with periodic boundary conditions. The MD simulations were performed at different temperatures (1500, 2000, 2500 and 3000 K) with canonical (NVT) ensemble. At the beginning of each simulation, an ensemble of velocities was generated using a random number generator with the specified seed at the specified temperature. The ensemble of generated velocities corresponds to a gaussian distribution with a mean of 0.0 and a sigma scaled to produce the requested temperature in this work. Note that a higher temperature (1500–3000 K) is usually adopted in the ReaxFF MD simulation to visualize the pyrolysis process within a computationally affordable time [36]. The Nosé -Hoover thermostat with a damping constant of 100 fs is adopted to control the system temperature. The total simulation time for each sample is 400 ps with a timestep of 0.25 fs. All MD simulations were carried out using Large-scale Atomic/Molecular Massively Parallel Simulator (LAMMPS) software [37].

## 3. Results and Discussions

### 3.1. Analysis of Bond Dissociation Energy by DFT

To predict the bond dissociation order of styrene-butadiene rubber during pyrolysis and explore the effect of linking methods between different repeat units, we paired the four repeat units in styrene-butadiene rubber and generated 16 typical trimers (Figure 1), and the DFT calculation was used to study the bond dissociation energy (BDE) of C–C bonds. Besides, to involve the difference of chemical environments of repeating units and reduce the total number of trimers, the same repeat unit is used on the left and right sides in each trimer. The BDE determines the thermal stability of the bond. The higher the energy, the more stable the bond is. In the pyrolysis process, the bond with low BDE tends to break at first. 

The BDE can predict the bond dissociation order of the C–C bonds in the main chain during the pyrolysis process, which is crucial for the study of the product distribution. Table 1 shows the BDE of C–C bonds in the main chain, and the serial number of carbon atom corresponds one-to-one with the number in Figure 1. For the case of BDE with the difference of 1 kJ/mol, we believe that these two bonds have similar BDEs. The difference is due to the precision error in gaussian calculation, and such error is within the allowable range. As for a trimer, the carbon atoms at both ends have one more hydrogen atom than carbon atoms in the main chain, resulting in a larger BDE between the C atom at the terminal position and C atom attached to the main chain [38]. For example, the BDE of C1–C2 bond (428.6 kJ/mol) in the CDC trimer is greater than that of C3–C4 bond (416.6 kJ/mol). However, the terminal C atom accounts for a very small proportion in the main chain and can be ignored in the analysis. In addition, the ring opening energy of the benzene and the BDE of the double bond are usually quite large comparing to the BDE of the single bond. From the BDE analysis of the AAA trimer, the C–C bond broken probability on the main chain of styrene is comparable. Similarly, the BDE analysis of the BBB trimer shows that the C–C bond broken probability on the main chain is comparable, and as a result, it tends to generate the 1-butene (C_4_). From the BDE analysis of CCC and DDD trimers, the BDE distribution of the two is similar. Both *cis*-butadiene and *trans*-butadiene tend to break the C–C bond adjacent to the double bond, i.e., the C–C bond between the repeating units. By changing the linking method between the repeating units, it is known that the C–C bond adjacent to the double bond in the main chain is more difficult than the C–C bond adjacent to the benzene ring. For example, comparing the BDE in the ABA and BAB trimers, the BDE of C1–C2 bond (311.3 kJ/mol) in the ABA trimer is higher than the counterpart (290.0 kJ/mol) in the BAB trimer. 

The above results indicate that the benzene ring and the vinyl groups have great influences on the BDE of the C–C bond on the main chain, as the benzene ring or the C=C double bond and C are connected with single bonds to form a hyperconjugation effect [39,40]. Therefore, the BDE of the C–C single bond adjacent to the benzene ring or the C=C double bond is decreased, where the low BDE bond is easily broken during the pyrolysis process. And thus, the bond broken prefers to happen between the repeating units and results in the pyrolysis products of C_4_ and C_8_. Further observation of BDE reveals that the hyperconjugation effect of vinyl is stronger than that of phenyl, which makes the stability of the hyper-conjugated system stronger. The larger overall energy in the hyper-conjugated system leads to the smaller BDE of the neighboring C–C bond, and thus the C–C single bond adjacent to the benzene ring is more difficult to break than that bonded to the double bond. In addition, taking the ADA trimer as an example, the C–C single bonds on the main chain are subjected to one hyperconjugation effect or two hyperconjugation effects, respectively. It is obvious that hyperconjugation effects are superimposed, resulting in that the BDE of the C–C bond affected by two hyperconjugation effects is significantly lower than that influenced by one hyperconjugation effect, for instance, the BDE of the C6–C7 bond is significantly lower than that of the C1–C2 bond. Similarly, for the CCC, CDC, DCD, and DDD trimers, the C–C bonds between the repeat units are subjected to two hyperconjugation effects and thus have low BDEs, which tend to break at first during the pyrolysis. 

### 3.2. Pyrolysis Product Distributions of Homopolymers via ReaxFF MD

**Figure 2:**
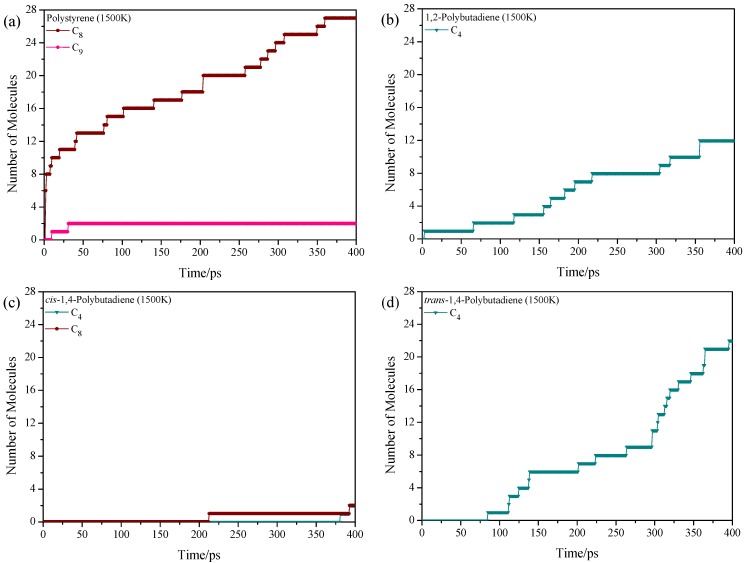
Time evolution of pyrolysis product distributions of homopolymers at 1500 K, (**a**) polystyrene, (**b**) 1,2-polybutadiene, (**c**) *cis*-1,4-polybutadiene and (**d**) *trans*-1,4-polybutadiene.

Through the analysis of the BDE, we can clearly know how the styrene-butadiene rubber macromolecules are decomposed into small molecules in the early stage of pyrolysis. However, the pyrolysis is a very complicated process because decomposition and reconnection are alternated, and thus the product distribution prediction cannot just rely on the BDE. To explore the pyrolysis product distribution directly and compare to corresponding experiments, ReaxFF MD is adopted to show the dynamic pyrolysis process at various temperatures. 4 homopolymer samples are built based on 4 kinds of repeat units, which are polystyrene, 1,2-polybutadiene, *cis*-1,4-polybutadiene, and *trans*-1,4-polybutadiene. Each sample consists of 4 polymer chains with 30 monomers in a chain, and therefore, the total numbers of atoms in each sample are 1928 (polystyrene), 1208 (1,2-polybutadiene), 1208 (*cis*-1,4-polybutadiene) and 1208 (*trans*-1,4-polybutadiene), respectively. Starting from the initial density of 0.1 g/cm^3^, the ReaxFF MD simulations are then performed at temperatures of 1500, 2000, 2500 and 3000 K with NVT ensemble. Due to high computational cost, ReaxFF MD simulations can only be performed up to nano-seconds which require higher temperatures (1500–3000 K) than experimental temperatures to obtain meaningful results [41]. Notably, there are some strategies to perform ReaxFF MD simulations at experimentally accessible temperatures [42,43], which may be beyond the research scope of this work. In real tire rubbers, the polymer chains are cross-linked and entangled due to the vulcanization and long chain lengths, respectively. The pyrolysis process is influenced by these two effects, especially the cross-linked network. For example, the transfer of S–S cross-linked bond is of high research interest during the pyrolysis process due to the increasingly severe environmental pressure [44]. However, the effects of cross-linked bonds and entanglement are beyond the scope of this work. The main product distributions of these four samples at 1500 K are shown in Figure 2. During the industrial application, the pyrolysis products of waste tire are classified into several groups (e.g., char, C_1_–C_4_ gas, light oil, heavy oil) according to the number of C atoms, and thus we considered species like C_n_ instead of the specified C_n_H_m_. The C*_n_* represents organic molecules that contain *n* carbon atoms. The C_2_ represents all organic molecules or intermediates that contain 2 carbon atoms such as C_2_H_2_, C_2_H_4_, C_2_H_3_· and C_2_H_6_. The product with the total number less than 4% of the largest C*_n_* is not considered in the figure. The C–C single bond between monomers tends to break and results in the formation of C_4_ (butene) and C_8_ (styrene), this result is consistent with the experiment [45,46] and DFT calculation due to the hyperconjugation effect. The 1500 K is actually much higher than the experimental temperature, and it is proved by pyrolysis experiment of polystyrene that high temperature (783 K in experiment) promotes the formation of styrene monomer and suppresses the formation of styrene trimer [46]. The small amount of C_9_ in the early pyrolysis stage of polystyrene is due to the random bond break of the next neighboring C–C bond adjacent to the benzene (*β*-scission [47]). The *cis*-1,4-polybutadiene is more stable than the *trans*-1,4-polybutadiene. It concludes that the intermonomer bonds tend to break at relatively low temperatures (around 650 K in experiment) during the pyrolysis process.

**Figure 3:**
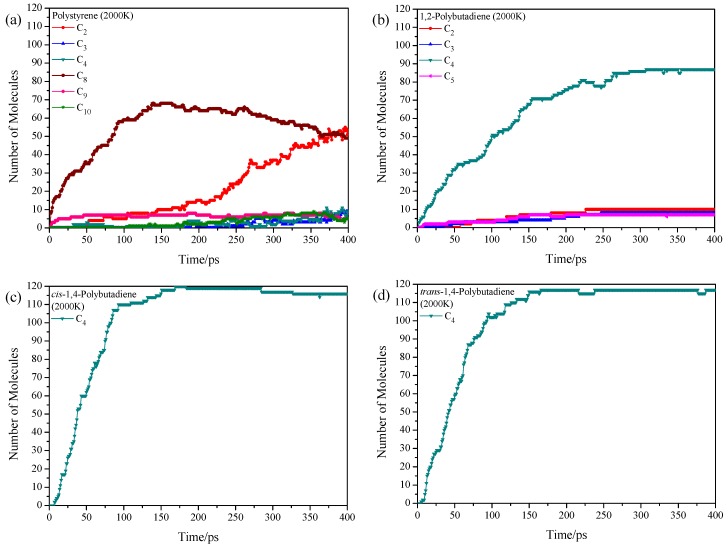
Time evolution of pyrolysis product distributions of homopolymers at 2000 K, (**a**) polystyrene, (**b**) 1,2-polybutadiene, (**c**) *cis*-1,4-polybutadiene and (**d**) *trans*-1,4-polybutadiene.

Higher pyrolysis temperature (above 700 K in experiment) usually results in higher gas fraction yield with expenses of the liquid fraction yield in experiments [6]. It means that the large fragments are further pyrolyzed into small ones with the increase of temperature. The product distributions for these 4 homopolymer samples at 2000 K show overall enhancements of total number of pyrolysis products (Figure 3). For the polystyrene, the temperature increase leads to the increase of C_8_ and C_9_, and the C_8_ increases sharply when the pyrolysis time is larger than 200 ps, accompanying with the formation of small fragments (e.g., C_2_, C_3_, and C_4_), while the C_6_ is not observed at this temperature. The bond break might still occur in the C–C single bond in the main chain instead of ring opening or *α*-scission. For the 1,2-polybutadiene, the main product is still the C_4_ (butene), and the C_2_, C_3_, and C_5_ are also found with small fractions (Figure 3b). It shows that the 1-butene structure is further pyrolyzed with enhanced temperatures. The pyrolysis situations for *cis*-1,4-polybutadiene and *trans*-1,4-polybutadiene are quite similar (Figure 3c,d), where only one main product (C_4_) is observed. Apparently, the temperature increase promotes the bond break between the monomers and stimulates the pyrolysis of intramonomer bonds.

**Figure 4:**
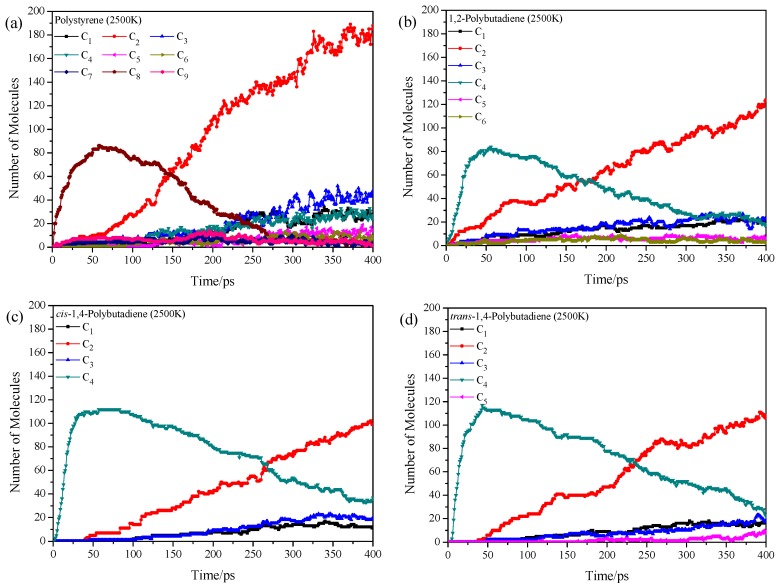
Time evolution of pyrolysis product distributions of homopolymers at 2500 K, (**a**) polystyrene, (**b**) 1,2-polybutadiene, (**c**) *cis*-1,4-polybutadiene and (**d**) *trans*-1,4-polybutadiene.

At 2500 K, the decomposition and reconnection are alternated, which result in the complicated product distributions. From the time evolution of the total number of pyrolysis products of homopolymers at different temperatures (Figure S6), higher temperature leads to more complicated product distributions. Figure 4 shows the product distributions at 2500 K. All samples generate several kinds of fragments due to the deep pyrolysis combining with the reconnection, where the final product is the C_2_ for all samples. The main pyrolysis products of polystyrene start from the C_8_. After around 150 ps, the C_8_ is decomposed into C_2_ and other small fragments. It means that the ring opening of benzene starts from the simulation temperature of 2000 K. The ring opening of benzene results in the diversification of pyrolysis products, where the number of product types are much more than those of the other three samples. The 1,2-polybutadiene, *cis*-1,4-polybutadiene, and *trans*-1,4-polybutadiene are all pyrolyzed into C_4_ at the very beginning, and then the C_4_ is further decomposed into C_2._ The *cis*-1,4-polybutadiene has the least number of product types (C_1_, C_2_, C_3_, and C_4_) among these 4 samples due to the most stable structure. The pyrolysis situation at 3000 K (Figure S1 in the Supplementary Information) is similar to that at 2500 K, where the C_2_ tends to decompose into C_1_ at 3000 K. This results also agree with the experimental findings where the solid pyrolytic carbon black particles are favored during the tire pyrolysis at very high temperatures (around 873 K in experiment) [15,48].

### 3.3. Pyrolysis Product Distributions of Styrene-Butadiene Rubber via ReaxFF MD

The structure of SBR is based on the aforementioned 4 kinds of repeat units, and thus the pyrolysis process strongly depends on the microstructure of the chain. To examine the effect of linking methods on the product distribution, 6 block copolymers are constructed with different combinations of 2 kinds of repeat units in each copolymer. Taking the A and B repeat units-based copolymer for example, the sample consists of 4 chains with 30 monomers (6 segments) in each chain. Each segment has 5 monomers and only one kind of repeat unit (A or B). And these A or B-based segments are alternately arranged. SBR applied in the tire rubber is crosslinked with sulfur and reinforced with carbon black [44], while the breakage of the crosslinked network of SBR not considered in this work. The initial density of these copolymer samples is set to 0.1 g/cm^3^, and then the ReaxFF MD simulations are performed at temperatures of 1500 (Figure S2), 2000 (Figure S3), 2500 (Figure S4) and 3000 K (Figure S5) with NVT ensemble. Generally, additive effects are observed in the pyrolysis product distribution, but it is possible to know the order of bond break under the competition between different repeat units. For example, the pyrolysis of polystyrene is suppressed when it is adjacent to trans-1,4-polybutadiene at 1500 K (Figure S2a,c). Similarly, comparing Figure 3b and Figure S3d, the 1,2-polybutadiene is not further decomposed into C_2_ when it is connected to cis-1,4-polybutadiene. The pyrolysis process at temperatures of 2500 K or 3000 K (Figures S4 and S5) is complicated, but the main product is still the C_2_ which agrees well with the results in homopolymers.

**Figure 5:**
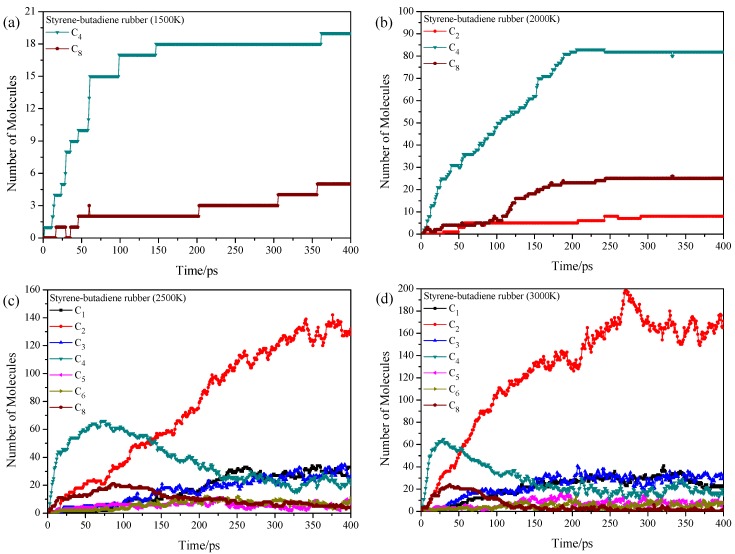
Time evolution of pyrolysis product distributions of styrene-butadiene rubber at different temperatures, (**a**) 1500 K, (**b**) 2000 K, (**c**) 2500 K and (**d**) 3000 K.

Based on the pyrolysis results of multiblock copolymers, the SBR chain is built with 4 kinds of repeat units and 32 monomers (8 segments). The A, B, C or D-based segments are alternately arranged. The SBR sample consists of 4 chains (1480 atoms in total) with the initial density of 0.1 g/cm^3^. Note that various kinds of microstructure of SBR can be found in experiment, where the property is strongly influenced by the structure. Only one artificial structure of SBR is adopted in this work. Figure 5 shows pyrolysis products of styrene-butadiene rubber polymers at 1500, 2000, 2500 and 3000 K with NVT ensemble. The C_4_ and C_8_ are observed at 1500 K which might be decomposed from polybutadiene and polystyrene, respectively. In the pyrolysis experiment, the C_4_ is the major product of polybutadiene [45]. Besides, the selectivity of primary product of polystyrene in experiment follows the trend of monomer (C_8_) > trimer > dimer, and high temperature promotes the formation of monomer at the expense of trimer [46]. With the increase of temperature, the C_2_ is appeared due to the fragmentation of large segments. At high temperatures (2500 and 3000 K), the C_2_ is increasing sharply with the decease of the C_4_ and C_8_. Combining with the results of multiblock copolymers, it concludes that the product distribution is possible to modulate via the variation of microstructure of polymer chains.

### 3.4. Effect of Initial Density on Pyrolysis Product Distributions

The pressure increase on pyrolysis leads to more viscous liquid products as well as more secondary reactions [49]. To examine the pressure effect on the pyrolysis product distribution of SBR, three SBR samples are prepared with different initial density via modulating the simulation box size of samples in Figure 5. The initial densities are set as 0.05, 0.20 and 0.50 g/cm^3^, respectively.

**Figure 6:**
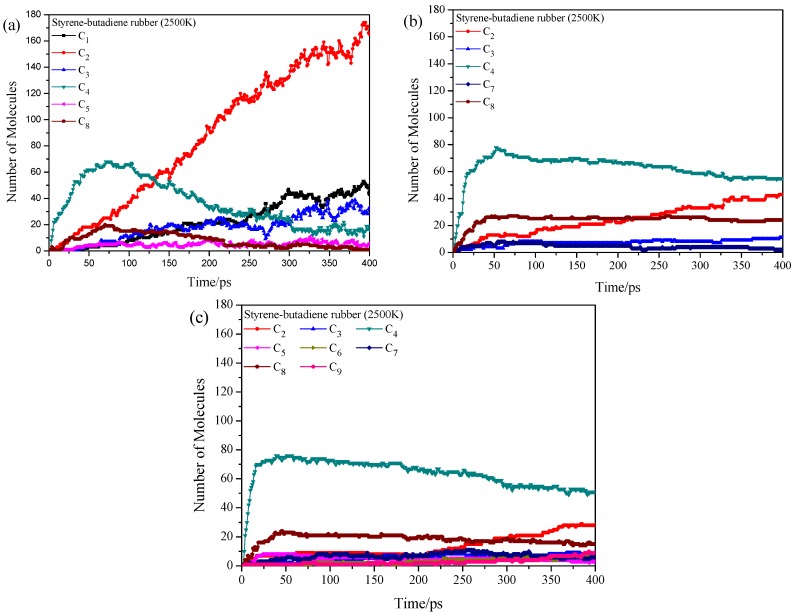
Time evolution of pyrolysis product distributions of styrene-butadiene rubber at different initial densities, (**a**) 0.05 g/cm^3^, (**b**) 0.20 g/cm^3^ and (**c**) 0.50 g/cm^3^.

With the help of ReaxFF MD, pyrolysis products of styrene-butadiene rubber polymers at three densities and the total number of pyrolysis products are shown in Figure 6. For the sample with density of 0.05 g/cm^3^, the product distribution is similar to that in Figure 5c (density of 0.10 g/cm^3^), though the number of small fragments (e.g., C_2_ and C) is increased. When the initial density increases to 0.20 g/cm^3^, the number of small fragments (e.g., C_2_ and C) is decreased accompanying with the decrease of total product type. The pyrolysis process occurs under the same volume, and thus the chemical equilibrium moves to the direction with the volume decrease when the pressure is enough high. It means that the decomposition and reconnection happen simultaneously. For the sample with density of 0.50 g/cm^3^, the pressure is even higher than previous samples, and more products are observed due to the occurrence of secondary reactions. The large segments such as the C_9_ increase with the pyrolysis time. These results agree with the experimental results that lower operating pressures can reduce the incidence of secondary reactions [6].

**Figure 7:**
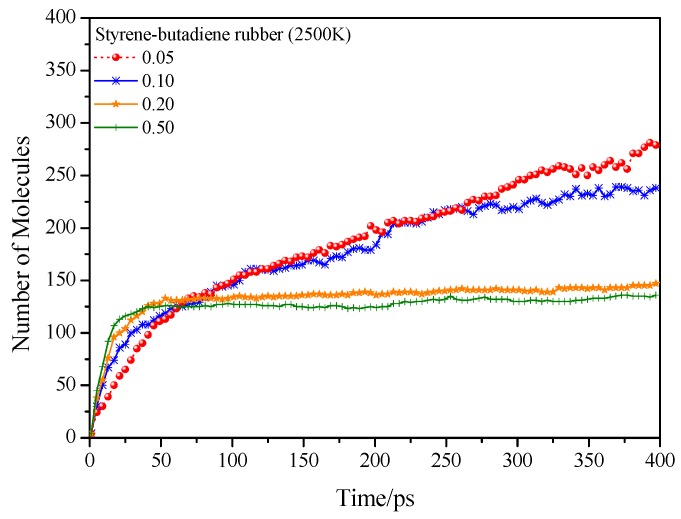
Time evolution of total number of pyrolysis products of styrene-butadiene at 2500 K with different initial densities.

From the variation of total number of pyrolysis products (Figure 7), the total numbers for low density samples (0.05 and 0.10 g/cm^3^) increase with the increase of simulation time, it meets to the tendency that the large segment fragments into many small ones. While the total numbers for high density samples (0.20 and 0.50 g/cm^3^) reach a plateau with the increase of simulation time, and one could imagine that the chemical equilibrium plays an important role during this process. Actually, the formation of main products and the product distribution are only slightly influenced by different initial aggregation structures (Figure S7). These product distributions are mainly controlled by reaction environments (e.g., temperature, pressure) and chemical structures of polymer chains.

## 4. Conclusions

The DFT and ReaxFF MD are used to study the pyrolysis of styrene-butadiene rubber. The DFT calculation is performed on several structural segments with different compositions to obtain bond dissociation energies. The double bonds in butadiene and ring opening in styrene monomers are more difficult than the bond breaking of single bonds during the pyrolysis process. Notably, due to the hyperconjugation effect, the next nearest single bonds connected with double bond or benzene usually prone to break with low dissociation energies. The linking methods between monomers have a slight influence on the values of bond dissociation energy. These DFT results are verified from the experimental studies reported in the literature. The following MD simulation is based on the chain segments to analyze the pyrolysis product distribution at different temperatures of 1500 K, 2000 K, 2500 K and 3000 K. The intermonomer bonds tend to break at relatively low temperatures (around 650 K in experiment) prior to intramonomer bonds, which result in the emergence of C_4_ (styrene monomer) and C_8_ (butadiene monomers). This decomposition order is corresponding to the DFT results and experiment findings [45]. With the increase of temperature, the large fragments are further pyrolyzed into small ones (e.g., C_2_ and C) due to the breaking of intramonomer bonds. The product types from pyrolysis of 1,2-polybutadiene or polystyrene segments are more than that of 1,4-polybutadiene segments due to the large BDE of nearest neighbor bonds adjacent to double bonds. In addition, the aromatic ring opening in styrene results in the complicated compositions of pyrolysis products. Our study shows the product distribution of styrene-butadiene rubber in various reaction environments and paves the way for the future studies in this area by offering an effective strategy.

## Figures and Tables

**Table 1:** The C–C bond dissociation energy (kJ/mol) in the main chain of trimers.

Trimer	C1–C2	C2–C3	C3–C4	C4–C5	C5–C6	C6–C7	C7–C8	C8–C9	C9–C10	C10–C11	C11–C12
CCC	429.4	711.8	420.7	221.4	420.5	712.7	420.5	220.6	420.7	711.8	429.4
CDC	428.6	715.6	416.6	233.1	421.2	719.9	422.4	236.7	416.7	716.4	428.6
DCD	434.1	718.9	424.4	232.5	417.1	715.5	415.9	235.3	424.6	721.0	435.7
DDD	435.9	721.3	426.2	235.4	421.0	719.6	423.2	236.1	421.5	719.9	434.0
CAC	427.5	714.0	409.6	229.3	300.0	292.1	413.0	716.3	428.1		
CBC	417.2	703.2	402.8	210.6	282.5	289.5	403.3	703.3	416.9		
DAD	435.8	720.2	417.3	231.7	299.5	294.2	420.3	720.8	435.6		
DBD	435.9	718.7	422.4	220.3	287.8	302.3	428.5	720.4	439.5		
ACA	316.8	300.1	413.8	713.9	412.9	235.9	319.1				
ADA	316.0	302.6	422.1	720.6	420.9	238.7	320.2				
BCB	305.7	303.0	413.2	713.6	414.9	223.5	306.1				
BDB	305.4	303.3	425.5	718.9	419.4	223.5	303.6				
AAA	317.0	307.0	301.9	300.5	316.0						
ABA	311.3	278.3	276.1	290.3	307.0						
BAB	290.0	283.0	281.4	277.7	296.3						
BBB	292.2	268.9	269.6	278.5	294.0						

## Supplementary Materials

The following are available online at https://www.mdpi.com/2073-4360/11/12/1967/s1, Figure S1: Time evolution of pyrolysis product distributions of homopolymers at 3000 K, (a) polystyrene, (b) 1,2-polybutadiene, (c) *cis*-1,4-polybutadiene and (d) *trans*-1,4-polybutadiene, Figure S2: Time evolution of pyrolysis product distributions of multiblock copolymers at 1500 K, (a) A and B-based segments, (b) A and C-based segments, (c) A and D-based segments, (d) B and C-based segments, (e) B and D-based segments and (f) C and D-based segments, Figure S3: Time evolution of pyrolysis product distributions of multiblock copolymers at 2000 K, (a) A and B-based segments, (b) A and C-based segments, (c) A and D-based segments, (d) B and C-based segments, (e) B and D-based segments and (f) C and D-based segments, Figure S4: Time evolution of pyrolysis product distributions of multiblock copolymers at 2500 K, (a) A and B-based segments, (b) A and C-based segments, (c) A and D-based segments, (d) B and C-based segments, (e) B and D-based segments and (f) C and D-based segments, Figure S5: Time evolution of pyrolysis product distributions of multiblock copolymers at 3000 K, (a) A and B-based segments, (b) A and C-based segments, (c) A and D-based segments, (d) B and C-based segments, (e) B and D-based segments and (f) C and D-based segments, Figure S6: Time evolution of the total number of pyrolysis products of homopolymers at different temperatures, Figure S7: Time evolution of pyrolysis product distributions of styrene-butadiene rubber with different equilibrium time at 400 K: (a) 0, (b) 15 ps, (c) 32.5 ps and (d) 50 ps, Table S1: The number of atoms in DFT and MD and the box size in MD.
