# Supplementary material for: Multiscale Simulation on Product Distribution from Pyrolysis of Styrene-Butadiene Rubber
Source: Polymers (Basel). 2019 Nov 29;11(12):1967. doi: 10.3390/polym11121967 (PMC6960849; doi:10.3390/polym11121967)
Supplement: Supplementary file 1 [file polymers-11-01967-s001.pdf]

Supplementary Information

Multiscale Simulation on Product Distribution from Pyrolysis of Styrene–Butadiene Rubber

Shengwei Deng [1*], Han Zhuo [1], Yinbin Wang [1], Shuai Leng [2*], Guilin Zhuang [1], Xing Zhong [1], Zhongzhe Wei [1], Zihao Yao [1] and Jian-guo Wang [1*]

[1] Institute of Industrial Catalysis, College of Chemical Engineering, State Key Laboratory Breeding Base of Green-Chemical Synthesis Technology, Zhejiang University of Technology, Hangzhou 310014, P. R. China.

[2] Qingdao Ecostar Intelligent Equipment Co., Ltd, Qingdao 266400, P. R. China.

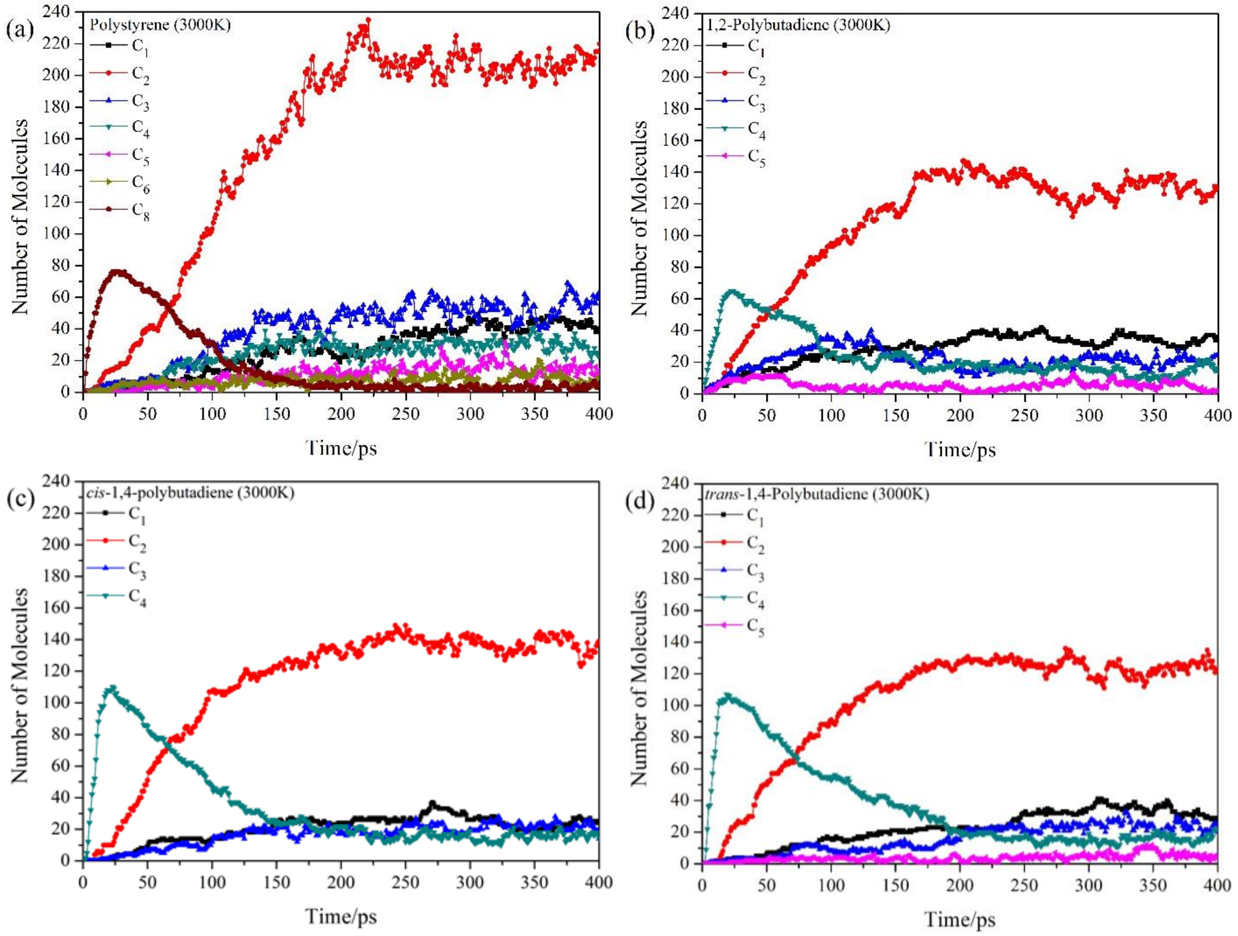

Fig. S1 Time evolution of pyrolysis product distributions of homopolymers at 3000 K, (a) polystyrene, (b) 1,2-polybutadiene, (c) *cis*-1,4-polybutadiene and (d) *trans*-1,4-polybutadiene.

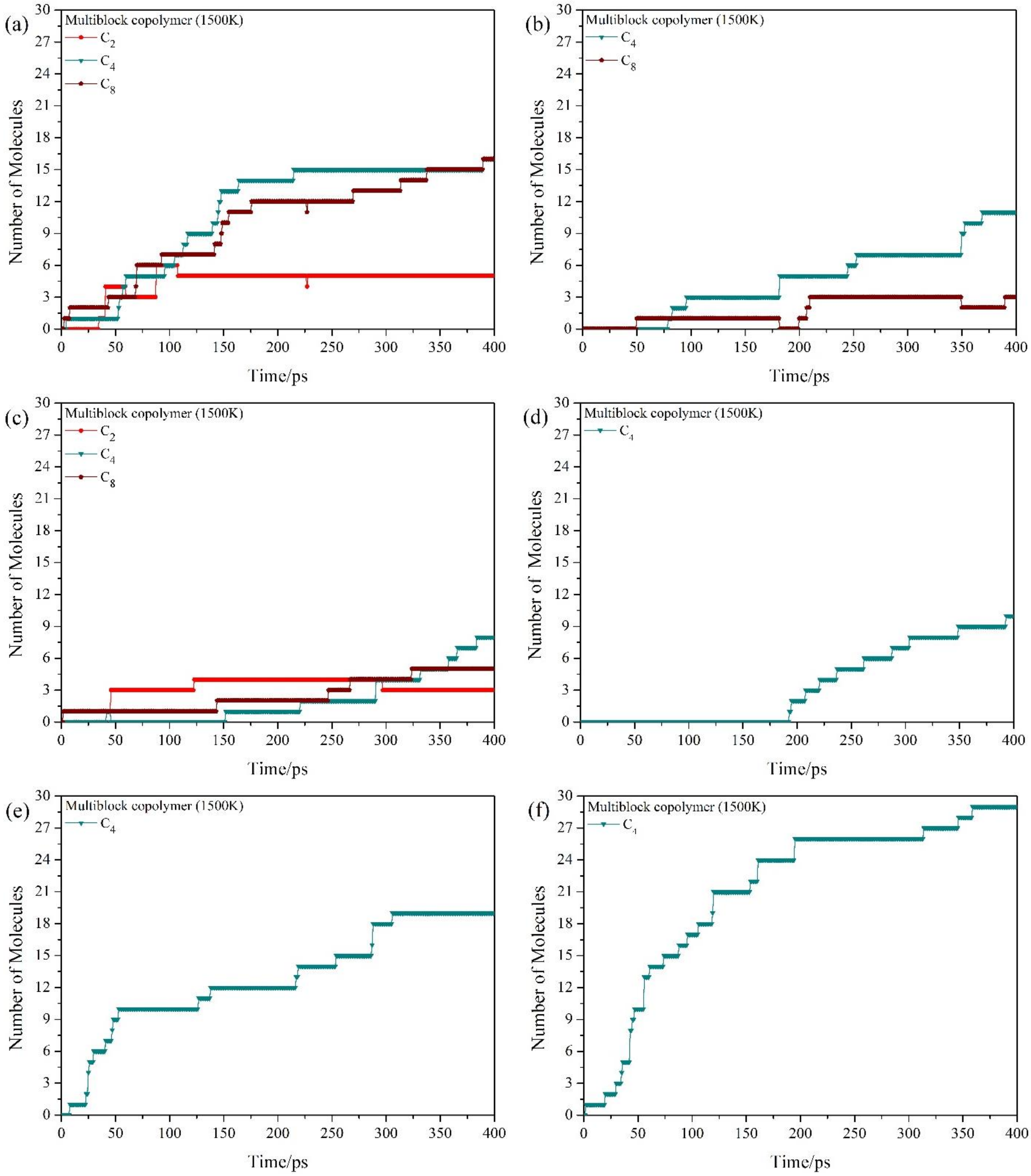

Fig. S2 Time evolution of pyrolysis product distributions of multiblock copolymers at 1500 K, (a) A and B-based segments, (b) A and C-based segments, (c) A and D-based segments, (d) B and C-based segments, (e) B and D-based segments and (f) C and D-based segments.

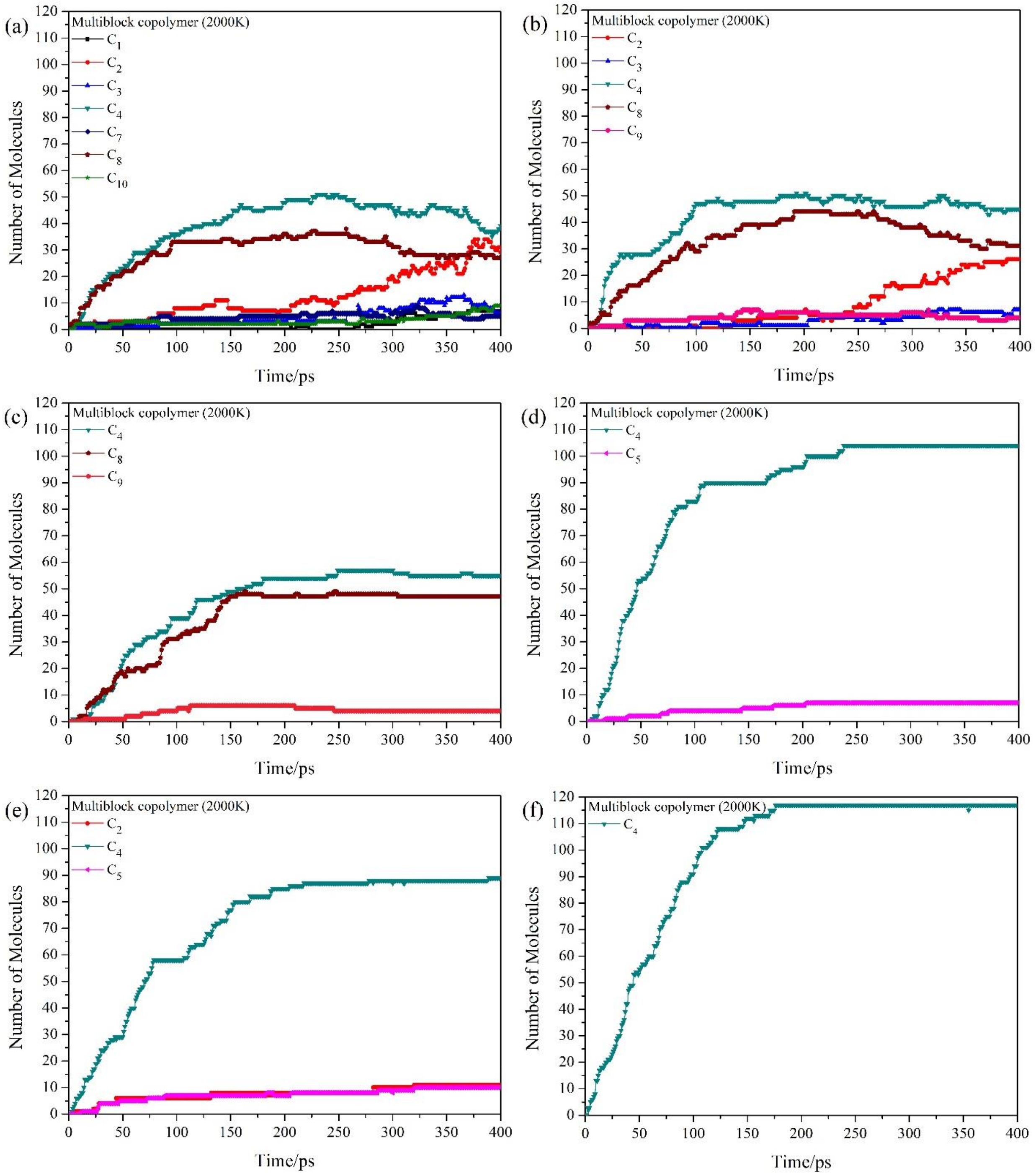

Fig. S3 Time evolution of pyrolysis product distributions of multiblock copolymers at 2000 K, (a) A and B-based segments, (b) A and C-based segments, (c) A and D-based segments, (d) B and C-based segments, (e) B and D-based segments and (f) C and D-based segments.

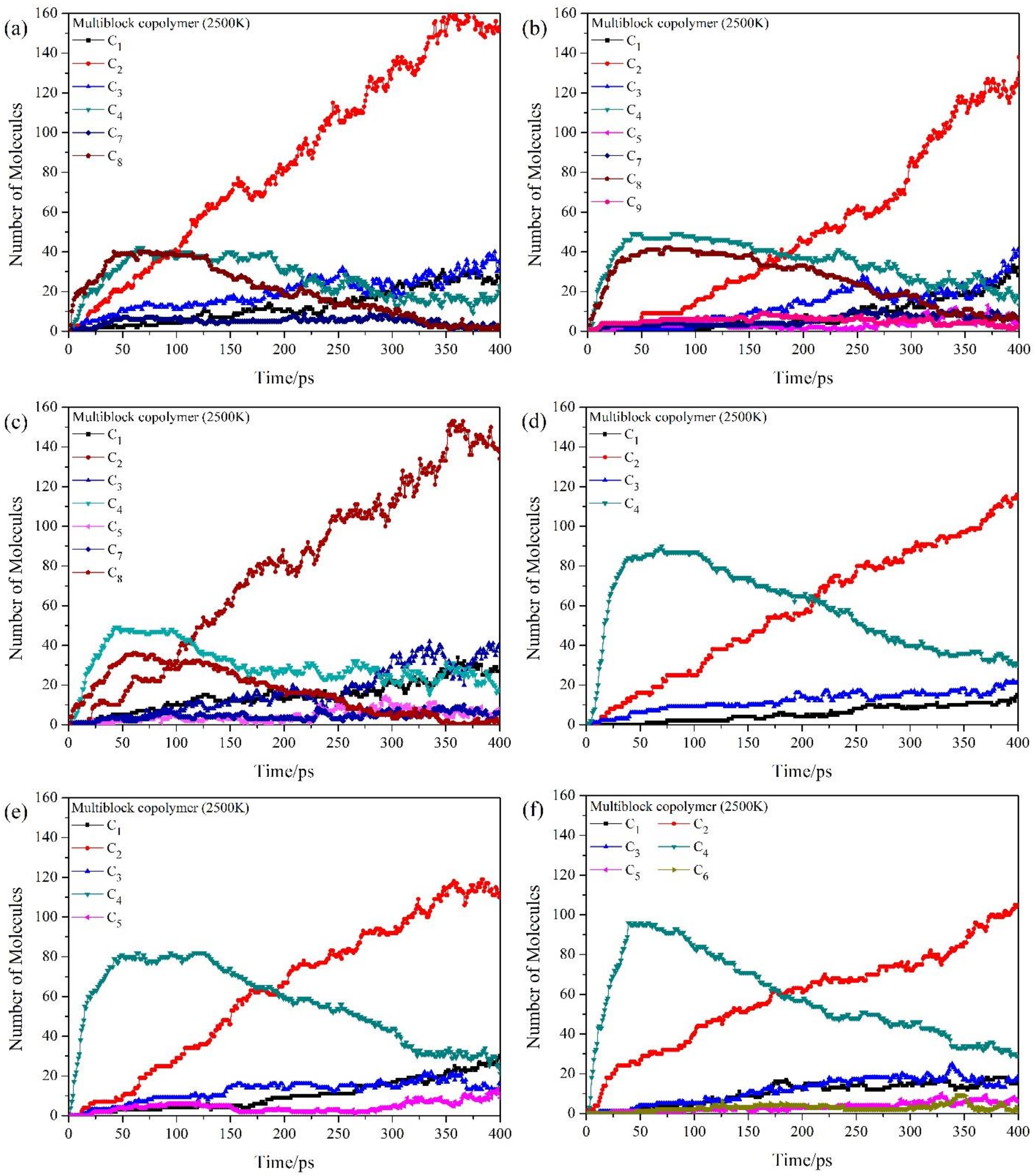

Fig. S4 Time evolution of pyrolysis product distributions of multiblock copolymers at 2500 K, (a) A and B-based segments, (b) A and C-based segments, (c) A and D-based segments, (d) B and C-based segments, (e) B and D-based segments and (f) C and D-based segments.

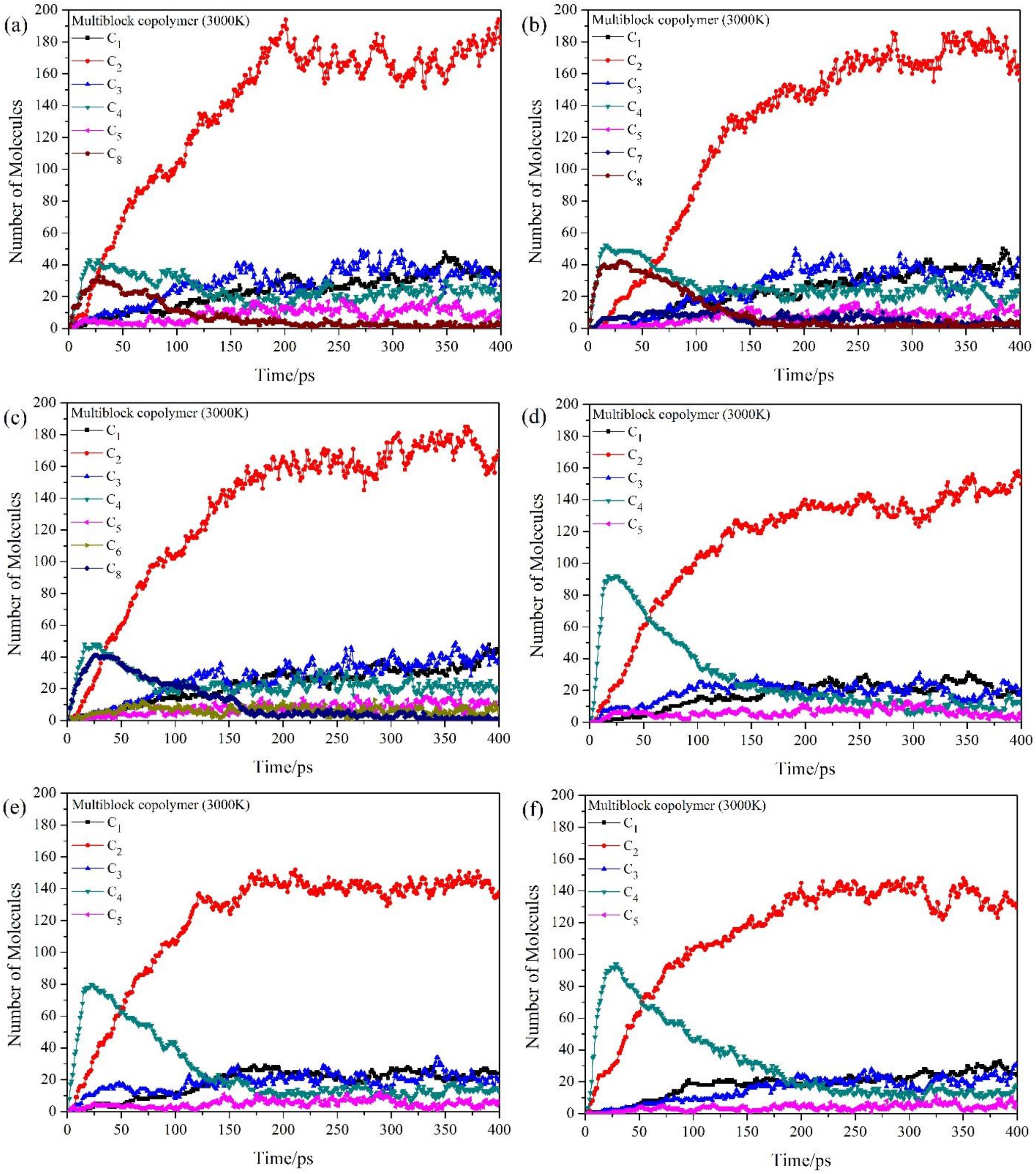

Fig. S5 Time evolution of pyrolysis product distributions of multiblock copolymers at 3000 K, (a) A and B-based segments, (b) A and C-based segments, (c) A and D-based segments, (d) B and C-based segments, (e) B and D-based segments and (f) C and D-based segments.

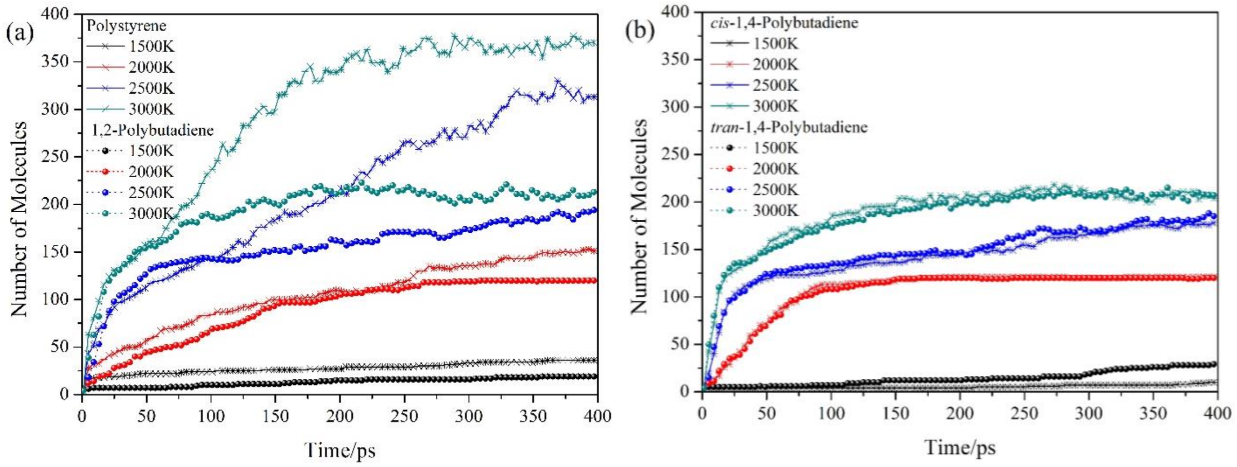

Fig. S6 Time evolution of the total number of pyrolysis products of homopolymers at different temperatures.
